# Asymptomatic Intestinal Malrotation Progressing to Midgut Volvulus in a Decompensated Alcoholic Cirrhotic Adult: A Rare Scenario Requiring Special Considerations

**DOI:** 10.1155/2020/4196012

**Published:** 2020-06-16

**Authors:** Vatche Melkonian, Pablo Quadri, Chintalapati R. Varma, Mustafa Nazzal, Henry B. Randall, Minh-Tri J. P. Nguyen

**Affiliations:** Division of Abdominal Transplant, Department of Surgery, Saint Louis University, Saint Louis, MO, USA

## Abstract

Intestinal malrotation usually presents in the pediatric population with midgut volvulus requiring emergency Ladd's procedure. Rarely, it remains asymptomatic and is discovered incidentally only during adulthood when it seldom causes intestinal complications. The scenario of a cirrhotic adult being diagnosed with asymptomatic intestinal malrotation with subsequent intestinal complications is thus extremely rare and to our knowledge has not been previously reported. We describe a 56-year-old man with decompensated alcoholic cirrhosis (Child-Pugh class C, MELD score 22) who was initially observed after an incidental diagnosis of intestinal malrotation on computed tomography. Observation continued as his liver disease improved with alcohol cessation (Child-Pugh class A, MELD score 8). He later presented with a closed loop bowel obstruction secondary to midgut volvulus at the time of alcohol relapse and liver redecompensation (Child-Pugh class C, MELD score 22-29). He underwent emergency Ladd's procedure during which his midjejunum was volvulized into an internal hernia space created by a thick Ladd's band containing large varices. The postoperative course was complicated by ileus and loculated bacterial peritonitis. Based on our experience, we discuss special considerations with regard to the surgical technique and timing of Ladd's procedure when encountering intestinal malrotation in a cirrhotic adult with portal hypertension.

## 1. Introduction

Intestinal malrotation is a congenital anomaly that is usually diagnosed in neonates or infants. When symptomatic, emergency Ladd's procedure is required to prevent a sequence of catastrophic intestinal events starting from midgut volvulus and leading to ischemic bowel, short-bowel syndrome, and death. In a minority of cases (<0.19%), intestinal malrotation remains asymptomatic or becomes diagnosed only in adulthood either acutely with midgut volvulus, chronically with intermittent bowel obstruction, or most frequently incidentally during imaging or surgery for other conditions [1]. The probability that a cirrhotic adult is diagnosed and suffers complications of intestinal malrotation is thus extremely low and to our knowledge has not previously been reported in the literature. We describe a decompensated alcoholic cirrhotic adult who presented with a closed loop small-bowel obstruction secondary to intestinal malrotation and discuss the special considerations with regard to the surgical technique and timing of Ladd's procedure in the presence of cirrhosis and portal hypertension.

## 2. Case Presentation

**Figure 1:**
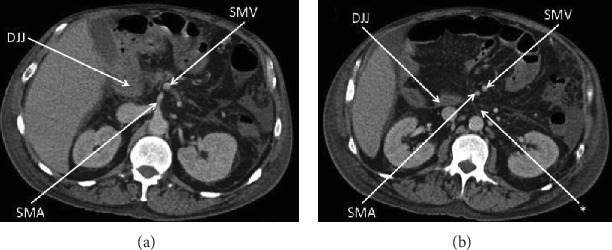
Abdominal computed tomography four years prior to current hospital presentation with incidental classic findings of intestinal malrotation. (a) Transverse plane at the second lumbar vertebral level demonstrating the duodenojejunal junction (DJJ) at the right of the midline and the superior mesenteric vein (SMV) to the left of the superior mesenteric artery (SMA). (b) Transverse plane at the third lumbar vertebral level further demonstrating the duodenojejunal junction (DJJ) at the right of the midline and the superior mesenteric vein (SMV) to the left of the superior mesenteric artery (SMA), as well as the absence of a retroperitoneal third portion of the duodenum (∗).

**Figure 2:**
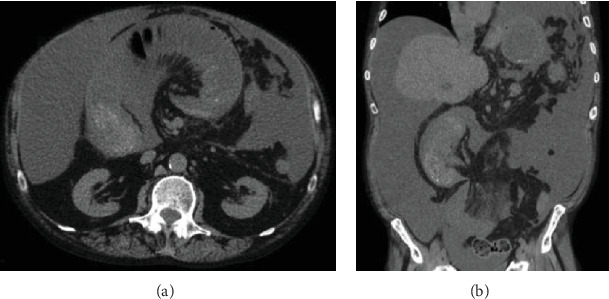
Abdominal computed tomography at the time of current hospital presentation in (a) transverse and (b) coronal planes demonstrating closed loop small-bowel obstruction in a known background of intestinal malrotation, cirrhosis, and ascites.

**Figure 3:**
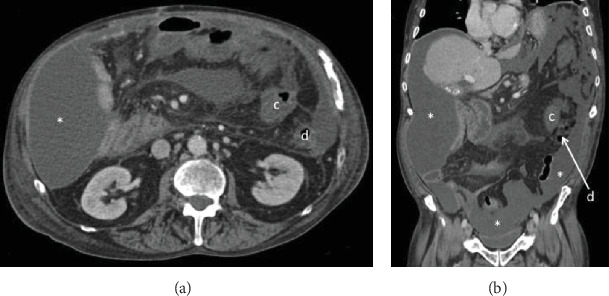
Abdominal computed tomography after Ladd's procedure in (a) transverse and (b) coronal planes demonstrating intraabdominal loculated ascites (∗) in Morrison's pouch, right and left paracolic gutters, and pelvis. There is resolution of small-bowel obstruction after Ladd's procedure with the cecum (c) positioned in the left upper quadrant medial to the descending colon (d).

The patient is a 56-year-old man with a history of umbilical hernia repair who was diagnosed four years ago with decompensated Child-Pugh class C alcoholic cirrhosis manifested by jaundice, encephalopathy, and large volume ascites requiring weekly paracentesis. His Model for End-Stage Liver Disease (MELD) score was 22. During an infectious work-up at the time, an abdominal computed tomography incidentally revealed intestinal malrotation with the classic findings of the duodenojejunal junction at the right of the midline, an abnormal position of the third portion of the duodenum, and an inversed relationship of the mesenteric vessels with the superior mesenteric vein to the left of the superior mesenteric artery (Figure 1) [2, 3]. There was also radiologic concern for partial small-bowel obstruction, but this did not translate clinically. The asymptomatic intestinal malrotation was thus expectantly managed. Three years ago, the patient's liver disease improved via alcohol abstinence and medical management. He no longer was encephalopathic or produced ascites. He was now Child-Pugh class A with a MELD score of 8 and underwent an open mesh repair of a painful right inguinal hernia without complications. Two years ago, the patient had alcohol abuse relapse and his cirrhosis progressively decompensated again with encephalopathy and large volume ascites. He was back at Child-Pugh class C with a MELD score ranging between 22 and 29. We were consulted on this patient when he now presented to the hospital with a two-day history of nausea, vomiting, and generalized abdominal pain. His pulse rate was 120 beats per minute and his blood pressure 100/70. His abdomen was distended and diffusely tender to palpation with a positive fluid wave test, but without peritoneal signs. He had leukocytosis of 30.8 thousand/*μ*L and lactic acidosis of 6.9 mmol/L. Diagnostic paracentesis of his ascites was negative for spontaneous bacterial peritonitis based on cell count. After fluid resuscitation and nasogastric tube decompression, he underwent an abdominal computed tomography which showed small-bowel dilation in the midabdomen associated with two transition points and a swirling appearance of the mesentery in the background of intestinal malrotation (Figure 2). These radiologic findings were concerning for a closed loop obstruction secondary to midgut volvulus. The patient was therefore brought emergently to the operating room for an exploratory laparotomy and Ladd's procedure. Upon entry into the peritoneal cavity, three liters of serous ascites was suctioned. The liver appeared grossly cirrhotic. A long segment of the midjejunum was dilated, ischemic, and volvulized into an internal hernia space created by a thick Ladd's band (right colon to duodenum) containing large retroperitoneal varices. The segment of the midjejunum was reduced from the internal hernia space and its mesentery untwisted in a counterclockwise fashion. Following wrapping in warm sponges, the midjejunum returned to a viable state and no intestinal resection was necessary. The thick Ladd's band containing retroperitoneal varices was then divided using a 36 mm jaw vessel-sealing device. Repositioning of the intestine to avoid volvulus resulted in the entire small bowel lying in the right lower quadrant and the cecum lying in the left upper quadrant. An appendectomy was performed to complete Ladd's procedure, and a closed suction drain was left in place for ascites control. Four units of packed red blood cells were transfused intraoperatively due to coagulopathy and extensive varices. The postoperative course was initially complicated by a systemic inflammatory response requiring transfer to the intensive care unit for vasopressor support and fluid resuscitation. A septic work-up including an abdominal computed tomography was negative apart from a positive intraoperative ascites culture. The patient improved with broad-spectrum intravenous antibiotics but then developed a prolonged ileus requiring nasogastric tube decompression and parenteral nutrition. He was discharged home on postoperative day 19 tolerating a regular diet and requiring weekly paracentesis. The pathology revealed an incidental 3 mm well-differentiated neuroendocrine tumor (grade 1, Ki − 67 < 3%) at the tip of the appendix that required no further treatment. The month following discharge was complicated by two readmissions to the hospital for loculated bacterial peritonitis without evidence of small-bowel obstruction recurrence on abdominal computed tomography (Figure 3). The loculated intra-abdominal fluid collections resolved with multiple ultrasound-guided drainages and broad-spectrum intravenous antibiotics.

## 3. Discussion

The above patient is a rare case of asymptomatic intestinal malrotation diagnosed in a cirrhotic adult that eventually progressed to midgut volvulus and required emergency Ladd's procedure. Diagnosis of intestinal malrotation was incidental and unsuspected on imaging, as the patient did not have a history of commonly associated congenital malformations such as duodenal atresia, gastroschisis, omphalocele, congenital diaphragmatic hernia, or heterotaxia [4]. In the context of cirrhosis and portal hypertension, we advocate the use of a vessel-sealing device instead of the usual use of electrocautery or scissors to safely divide the Ladd's band, which was thick and collateralized with large retroperitoneal varices instead of thin and avascular [5]. It remains controversial if prophylactic Ladd's procedure should be performed for asymptomatic intestinal malrotation beyond infancy. Most groups recommend elective Ladd's procedure in all patients to prevent catastrophic midgut volvulus while others advocate watchful waiting in adulthood as the risks of surgical complications outweigh the benefits of volvulus prevention beyond age 20 [6, 7]. The scenario in which a cirrhotic adult has asymptomatic intestinal malrotation however deserves special considerations with regard to prophylactic Ladd's procedure and its timing. In our patient, failure of expectant management resulted in emergency Ladd's procedure at the time when his liver disease was at its worst (Child-Pugh class C, MELD score between 22 and 29, large volume ascites). In retrospect, if we met this patient four years earlier, we would strongly have considered prophylactic elective Ladd's procedure during the time window when his liver disease was optimized (Child-Pugh class A, MELD score of 8, no ascites) for the following reasons. Complications after elective Ladd's procedure (22%) are significantly less than when performed emergently (66%) [1]. Mortality after elective nonhepatic abdominal surgery (9%) is also significantly less than when performed emergently (47%) in a cirrhotic patient [8]. Moreover, if emergency nonhepatic abdominal surgery is required, perioperative mortality in a cirrhotic patient with Child-Pugh class A or MELD score < 10 (0-20%) is less than that in a patient with Child-Pugh class C or MELD score > 15 (60-72%) [8]. The avoidance of intraoperative blood transfusion during nonhepatic abdominal surgery, which is likely more easily achievable during elective surgery in a compensated cirrhotic patient, is also significantly associated with less perioperative mortality (5% without transfusion compared to 43% with transfusion) in cirrhotic patients [8]. A laparoscopic approach, which is safe in cirrhosis [9] and in elective Ladd's procedure [7], could also have been attempted with the potential of reducing surgical complications and hospital stay.

In conclusion, intestinal malrotation is a rare occurrence in a cirrhotic adult. Utilizing a vessel-sealing device is useful in dividing varix-containing Ladd's band. Prophylactic elective Ladd's procedure when liver disease is optimized should be considered in order to avoid midgut volvulus at a time of liver decompensation and in order to minimize surgical complications, morbidity including peritonitis, and mortality.
